# Temperature Stress and Responses in Plants

**DOI:** 10.3390/ijms20082001

**Published:** 2019-04-24

**Authors:** Nobuhiro Suzuki

**Affiliations:** Department of Materials and Life Sciences, Faculty of Science and Technology, Sophia University, 7-1 Kioi-Cho, Chiyoda, Tokyo 102-8554, Japan; n-suzuki-cs6@sophia.ac.jp; Tel.: +81-3-3238-3884

Extreme temperatures can have detrimental effects on yield production worldwide. Despite extensive studies focusing on the mechanisms that regulate the responses of plants to temperature stresses, such agricultural problems have not been solved. Indeed, the establishment of strategies to enhance tolerance of plants and crops to extreme temperature is challenging, because of the high complexity in the underlying molecular and physiological mechanisms involving multiple pathways that are differently coordinated depending on growth stages, type of tissues, intensity, and duration of stresses, plant species, and so on.

This Special Issue, entitled “Temperature Stress and Responses in Plants” presents four reviews and five research articles highlighting responses of plants to high or low temperatures and their detailed regulatory mechanisms.

Three research articles and one review addressed responses of plants to low temperature. Zhang et al. revealed that reactive oxygen species (ROS) scavenging systems triggered by nitrate reductase (NR)-derived nitric oxide (NO) might be important for the protection of forage legumes against cold stress [1]. The significance of NO-dependent ROS scavenging systems was also supported by the finding that NO production and its derived antioxidant capacity were higher in a cold-tolerant legume (*Medicago falcate* L.) compared with a cold-sensitive one (*Medicago truncatula*). In contrast, Arfan et al. indicated the important role of ROS to trigger the NO signaling that regulates the alternative oxidase (AOX) pathway and photosystem II efficiency in brassinolide (BR)-dependent cold responses in *Medicago truncatula* [2]. The findings from these two studies raised two questions, “How is the hierarchy between ROS and NO regulated during cold stress?” and “How is the delicate balance between ROS production and scavenging modulated during cold stress?”. These questions could be addressed by the elucidation of Ca^2+^ signaling networks, because of its integration with ROS and NO regulatory systems under cold stress [3]. Yuan et al. proposed that Ca^2+^ signaling activated by cold sensing machinery on the plasma membrane facilitates ROS producing NADPH oxidases as well as many pathways including NO signaling [3]. Further studies are required to address how Ca^2+^ signaling is modulated depending on the cellular ROS level. In addition, it should be necessary to elucidate detailed mechanisms to sense ROS levels in cells during cold stress in future studies. In contrast to the studies above that mainly focused on the “activation” of mechanisms to protect plants against cold stress [1,2,3], Xu et al. demonstrated the strategy of plants to survive under cold stress by “abscission” of cellular metabolisms [4]. Quantitative proteome analysis using winter turnip rape (*Brassica rapa* L.) revealed that the accumulation of proteins involved in essential biological processes, such as photosynthesis and respiration, was lower in the cold-tolerant variety compared to the sensitive one under cold stress. Taken together, these studies suggested the significance of delicate balances between different mechanisms (i.e., ROS scavenging vs production, and activation vs abscission) in the regulation of cold responses in plants. Further studies are required to uncover the key signaling that switches different mechanisms.

Two research articles and three reviews addressed responses of plants to high temperature. Stewart et al. demonstrated alteration in the vascular organization pattern as well as enhanced expression of transcripts encoding C-repeat binding factors (CBFs) in Arabidopsis mutants with an abnormal cellular redox state under heat stress [5]. This research suggested possible links between the regulation of cellular redox state, maintenance of water transport, and acclimatory gene expression under heat stress. It can be expected that the links between these different processes under heat stress might be regulated by complex mechanisms, because ROS and redox regulatory systems constitute large networks with various other pathways including Ca^2+^, NO and plant hormone signals [6]. In addition, such signaling networks associated with ROS and redox regulatory systems are flexibly coordinated to tailor the heat stress responses depending on the types of heat stress, tissues, and growth stages [6]. Many studies have uncovered the flexible and complex signaling networks underlying heat responses in plants, and these findings raised a further question “How do plants sense the alteration of temperature and shift the state of their acclimatory mechanisms?”. A review presented by Susila et al. might provide important clues to address this question. This review summarized mechanisms of the temperature sensing and responses that regulate the timing of state transition from vegetative to reproductive growth [7]. Authors suggested that various processes including histone modification, DNA methylation, and the regulation of the circadian clock are flexibly modulated to sense temperature changes and accelerate or inhibit flowering via regulating the expression of floral activator and repressor genes. Furthermore, temporal differences in heat stress responses might be regulated by DNA methylation. Liu et al. showed that, compared to other eudicot plants, a relatively higher proportion of CHH methylation was detected in Chinese cabbage [8]. Authors also revealed that different sets of genes were methylated and upregulated during early and late stages of heat stress. Although these studies addressed detailed molecular and physiological mechanisms underlying heat stress responses in plants, researchers still need to further consider the strategies to utilize such useful information to improve situations of agriculture and ecology. Interestingly, a review presented by Sampaio Filho et al. explored the physiological adaptations of trees in forests to high temperature and drought [9]. In this review, the authors proposed that a large fraction of ABA might be produced in leaves through isoprenoid pathways in forests. In leaves, ABA signals might be directly integrated with carbon metabolism and ROS regulatory systems through the regulation of stomatal movement. 

We can suggest that the complexity of the molecular and physiological mechanisms might be essential for flexible responses of plants to fluctuating temperature under natural environments. We are confident that further understanding of such flexible temperature responses in plants can lead to an increase in yield production worldwide.

## Abbreviations

ROS	Reactive oxygen species
NR	Nitrate reductase
NO	Nitric oxide
AOX	Alternative oxidase
BR	Brassinolide
CBF	C-repeat binding factor
ABA	Abscisic acid
